# Management of New Post-Operative Arrhythmia in Cardiac Patients

**DOI:** 10.1186/1749-8090-10-S1-A137

**Published:** 2015-12-16

**Authors:** Michael MacPherson, Gwyn Beattie, Robyn Smith, John Butler

**Affiliations:** 1 Cardiothoracic Department, Golden Jubilee National Hospital, Greater Glasgow and Clyde, G81 4HX, UK

## Background/Introduction

Literature suggests 25-50% of patients develop arrhythmia following cardiac surgery. Within a regional cardiothoracic centre in Scotland, guidelines exist on initial management of post-operative atrial arrhythmia. However, in practice various approaches are used. Arrhythmias contribute to prolonged hospital stay and high dependency readmission.

## Aims/Objectives

To assess staff awareness of local guidelines, audit the proportion of patients developing arrhythmia and check for correlation with logistic EuroScore and length of hospital stay. Approach to management of arrhythmia within the initial 48 hours following identification was compared to local guidelines.

## Method

Staff questionnaire was circulated prior to a four week data collection period. Consecutive cases of new post-operative arrhythmia were identified within the intensive care unit, high dependency unit and cardiac ward. Contributions were made to the proforma by anaesthetic, cardiology and surgical consultants.

## Results

Results from the staff questionnaire indicated 64% were unaware of local guidelines. 25 cases were identified; 2 in ICU, 15 in HDU and 8 on the ward. Case mix included CABG (37%), valve replacement (20%), mixed CABG and valve (23%), and other (20%). Average logistic EuroScore was 4.34%, higher than the centres average. In the majority of cases (56%), onset of arrhythmia occurred 24-72 hours following surgery. Arrhythmia most frequently identified was atrial fibrillation (22 cases). There was one case each of SVT, NSVT and atrial flutter. During analysis of atrial fibrillation cases, when asking 'would management have differed if local guidelines were adhered to?', the answer was yes in 8 (36%) of cases. In the majority of cases arrhythmia lasted less than 48 hours. 2 cases were cardioverted. Duration of inpatient stay for this study group was 9.6 days, significantly longer than the centre's average of 6 days.

## Discussion/Conclusion

There is not a definite treatment strategy for the management of new onset atrial arrhythmias following cardiac surgery. Management most frequently included potassium and magnesium replacement, oral beta blocker and IV amiodarone. This project raises the question as to whether a standardised guideline is feasible in practice. Complexities include variation in patient suitability for management and consultant preferences.

